# Cutaneous Perineurioma of the Medial Superciliary Arch: An Uncommon Location for a Rare, Benign Spindle Cell Neoplasm

**DOI:** 10.7759/cureus.103023

**Published:** 2026-02-05

**Authors:** Sri Naidnur, Valeria González-Molina, Kara Asbury, Emily DeSantis, Rick Lin

**Affiliations:** 1 Dermatology, Oasis Dermatology Group, McAllen, USA; 2 Department of Dermatology, HCA Healthcare Corpus Christi Medical Center - Bay Area, McAllen, USA; 3 Dermatopathology, Sagis Diagnostics, Houston, USA

**Keywords:** benign skin tumor, benign tumor, cd34, cutaneous perineurioma, dermatology, dermatopathology, ema, s100, soft tissue perineurioma

## Abstract

Cutaneous perineuriomas are rare, benign peripheral nerve sheath tumors that are often mistaken for other adnexal or spindle cell lesions, with facial involvement being uncommon. Recognition is important to ensure accurate diagnosis and avoid unnecessary overtreatment.

We report a case of a 36-year-old Hispanic woman with a slowly enlarging papule of the left medial superciliary arch, histologically and immunophenotypically confirmed as a cutaneous perineurioma. This case highlights the clinicopathologic features, immunohistochemical profile, and management considerations of cutaneous perineurioma arising in a rare, cosmetically sensitive facial location, adding to the limited body of published cases.

## Introduction

Perineuriomas are uncommon, benign soft tissue neoplasms derived from perineurial cells, which form the perineurium surrounding peripheral nerve fascicles [1]. They are broadly classified into intraneural and extraneural types, the latter including soft tissue and cutaneous variants [1]. Cutaneous perineuriomas typically present as small, slow-growing dermal papules or nodules and are frequently misdiagnosed as other benign cutaneous lesions [1,2].

The most common sites are the trunk and extremities, while facial involvement is distinctly uncommon [1]. Although perineuriomas have been described in the literature, cutaneous cases involving the face are uncommon, and their rarity, combined with the limited number of published case reports and small case series, precludes reliable percentage-based estimates of facial involvement. Management is individualized based on clinical and anatomic factors, as no standardized treatment or surveillance guidelines exist [3]; recurrence following complete excision is rare [1,2]. Misdiagnosis may lead to unnecessary aggressive treatment when perineurioma is mistaken for more aggressive spindle cell neoplasms, particularly in cosmetically sensitive areas such as the face, underscoring the importance of accurate clinicopathologic correlation. We present a case of a cutaneous perineurioma arising in the left medial superciliary arch.

## Case presentation

A 36-year-old Hispanic woman with no significant past medical history presented with a skin-colored to slightly erythematous papule on the left medial superciliary arch (medial eyebrow) that had been present for approximately two years. The lesion was initially perceived by the patient as a “pimple” and gradually increased in size. The patient attempted superficial removal with a razor blade on two occasions, resulting in incomplete removal and persistence of the lesion. The patient denied similar lesions elsewhere, preceding trauma, systemic symptoms, or relevant comorbidities.

**Figure 1:**
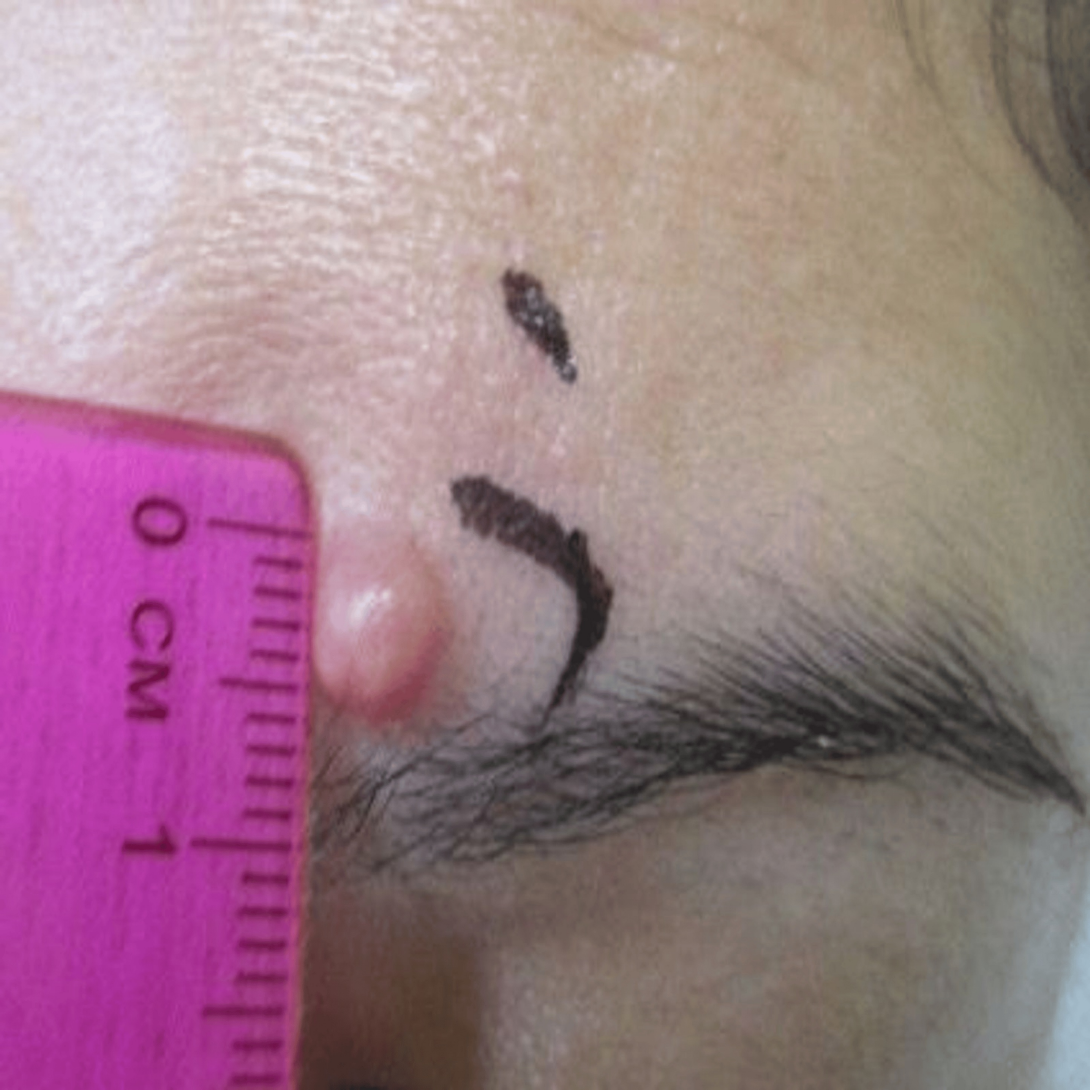
Clinical photograph demonstrating a skin-colored to slightly erythematous papule on the left medial superciliary arch, marked for shave biopsy.

The lesion was intermittently pruritic and occasionally associated with a mild tingling sensation, but was otherwise asymptomatic. Physical examination revealed a soft, well-circumscribed papule measuring less than 1 cm on the left medial eyebrow, without regional lymphadenopathy (Figure 1).

A shave biopsy was performed. The initial clinical differential diagnosis included pyogenic granuloma, amelanotic melanoma, hidrocystoma, and neurofibroma. Histopathologic evaluation demonstrated a dermal spindle cell proliferation measuring 0.7 × 0.6 × 0.3 cm, consistent with a perineurioma. The diagnosis was rendered by the primary dermatopathologist and subsequently confirmed in consultation with an external academic dermatopathologist at the Cleveland Clinic.

**Figure 2:**
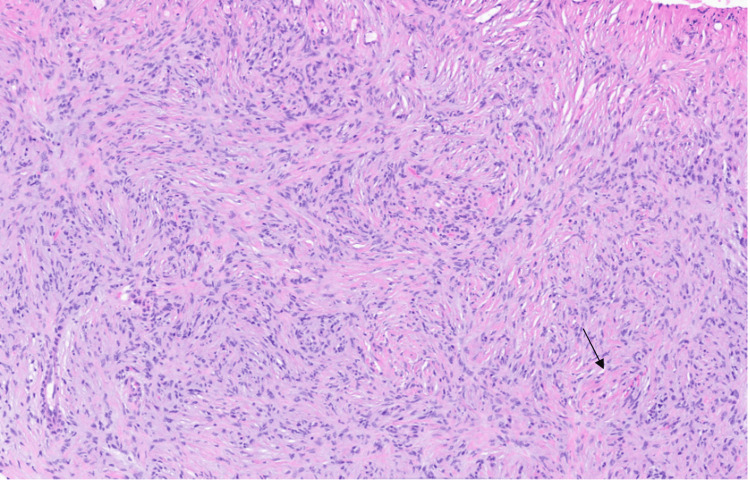
Low-power H&E-stained section demonstrating bland spindle cells arranged in short fascicles with a whorled architectural pattern within the dermis (original magnification ×4). The arrow highlights a representative whorled pattern.

**Figure 3:**
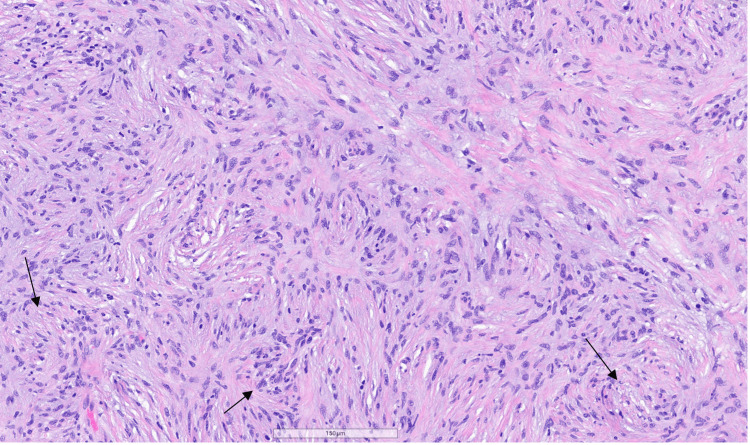
H&E section showing bland spindle cells with elongated nuclei arranged in a whorled, short-fascicular pattern within a collagenous stroma (magnification ×10). Arrows highlight areas of whorled architecture.

Microscopic examination of hematoxylin and eosin (H&E)-stained sections revealed a proliferation of bland spindle cells arranged in short fascicles with a whorled growth pattern within the dermis (Figure 2). The cells exhibited ovoid to slightly wavy nuclei with vesicular chromatin and mild nuclear size variability (Figure 3), without mitotic activity or cytologic atypia.

**Figure 4:**
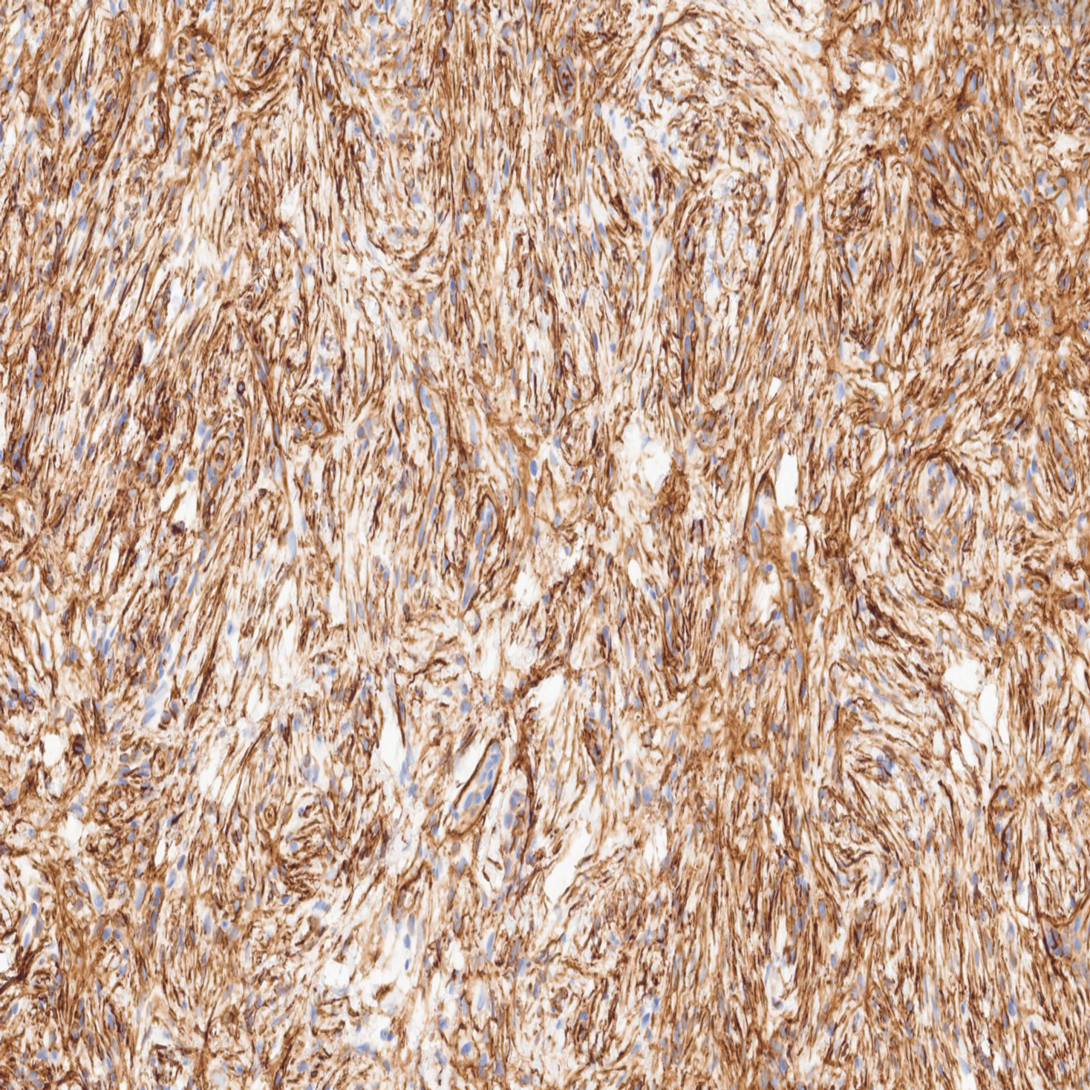
CD34 immunohistochemical stain showing strong positivity within the lesional spindle cells.

**Figure 5:**
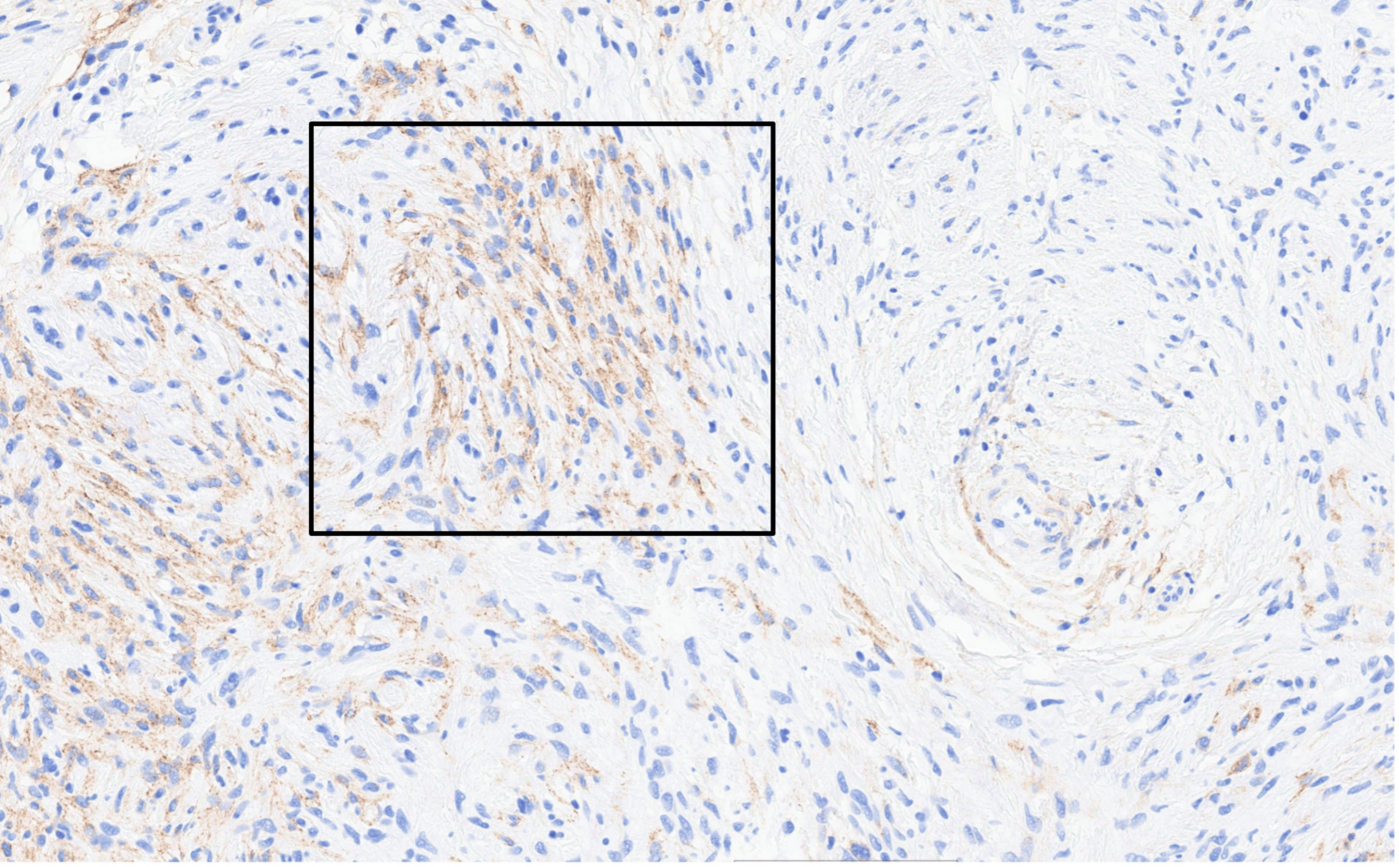
Epithelial membrane antigen (EMA) immunohistochemical stain demonstrating membranous positivity within lesional spindle cells. The box highlights an area of representative EMA expression.

**Figure 6:**
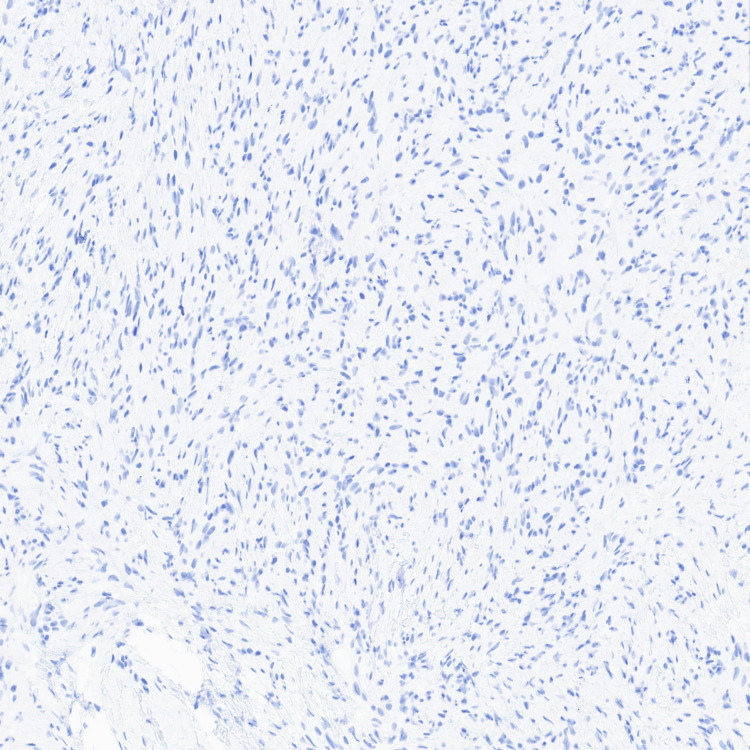
S100 immunohistochemical stain demonstrating negative staining in the spindle cell proliferation.

Immunohistochemical studies demonstrated strong CD34 positivity in lesional cells (Figure 4). Scattered positivity for epithelial membrane antigen (EMA) (Figure 5) and smooth muscle actin (SMA) was observed, while NKI/C3 highlighted scattered lesional cells, supporting perineurial differentiation. In contrast, S100 (Figure 6), MART-1, and CD10 were negative, thereby excluding Schwann cell-derived, melanocytic, and fibrohistiocytic neoplasms. These findings supported the diagnosis of a cutaneous perineurioma.

**Figure 7:**
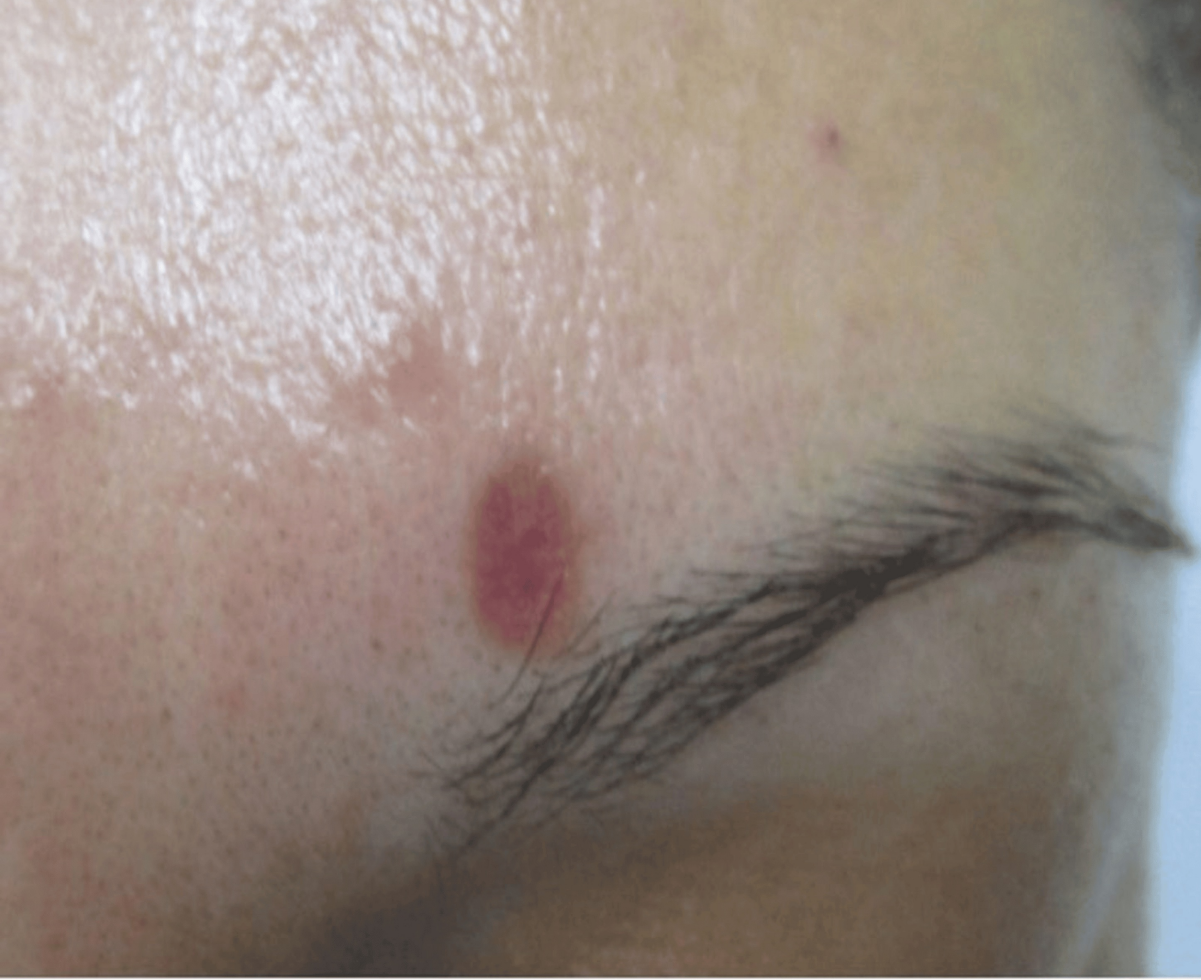
Post–shave biopsy photograph of the left medial eyebrow demonstrating mild scarring and post-inflammatory hyperpigmentation without a residual lesion.

At follow-up, post-biopsy examination revealed mild scarring and post-inflammatory hyperpigmentation at the biopsy site, without a palpable residual lesion (Figure 7). Management options, including complete surgical excision versus clinical observation, were discussed. Given the benign nature of the tumor, absence of residual lesion, and cosmetic considerations, the patient elected clinical monitoring with planned re-evaluation in three to six months.

## Discussion

Perineuriomas should be distinguished from other spindle cell neoplasms, including schwannomas, neurofibromas, dermatofibromas, and spindle cell melanocytic lesions [1]. Accurate diagnosis is important, as management and prognostic considerations differ among these entities. Unlike Schwann cell-derived tumors, which demonstrate diffuse S100 positivity, perineuriomas typically exhibit EMA positivity with absent S100 staining, reflecting their perineurial origin [3,4]. While S100 negativity is not specific, its absence in conjunction with EMA positivity supports perineurial differentiation [1,3,4].

In contrast, CD34 is a nonspecific marker that may be diffusely expressed in other spindle cell neoplasms, such as solitary fibrous tumors and dermatofibrosarcoma protuberans, and demonstrates variable expression in perineuriomas [2,4,5]; therefore, CD34 expression must be interpreted in conjunction with the overall histopathologic features on H&E and the broader immunophenotypic profile.

From a diagnostic standpoint, cutaneous perineuriomas characteristically demonstrate bland spindle cells arranged in whorled fascicles with minimal cytologic atypia or mitotic activity, features that help distinguish them from more aggressive spindle cell neoplasms [1,4]. These findings were present in our case and supported the benign nature of the lesion.

Local recurrence is uncommon following complete surgical excision [5,6]. In this patient, recurrence after attempted self-removal was likely due to incomplete elimination of the dermal component rather than true biologic recurrence, underscoring the importance of definitive excision and appropriate patient counseling regarding superficial removal techniques.

Long-term in-person follow-up was not available in this case; however, given the benign behavior of cutaneous perineurioma, care was guided by clinicopathologic findings. No standardized surveillance guidelines exist for cutaneous perineuriomas, and follow-up is therefore individualized [3]. In cosmetically sensitive locations such as the face, management should balance definitive treatment with aesthetic considerations and patient preference. In select cases, particularly when lesions are asymptomatic, small, or without residual perineurioma following biopsy, clinical observation may be reasonable, with surgical excision reserved for recurrence or cosmetic concerns. Taken together, this case highlights the importance of clinicopathologic correlation and shared decision-making in the management of facial cutaneous perineuriomas.

## Conclusions

Cutaneous perineuriomas are rare, benign spindle cell tumors that may clinically mimic adnexal or fibrohistiocytic lesions. Recognition of their characteristic histologic and immunophenotypic profile, particularly EMA positivity with S100 negativity, is essential for accurate diagnosis. Complete excision is typically curative, and recurrence is rare. In the absence of standardized follow-up guidelines, management should be individualized based on clinical findings, lesion location, and patient preference.
